# Lumbar segmental instability: a criterion-related validity study of manual therapy assessment

**DOI:** 10.1186/1471-2474-6-56

**Published:** 2005-11-07

**Authors:** J Haxby Abbott, Brendan McCane, Peter Herbison, Graeme Moginie, Cathy Chapple, Tracy Hogarty

**Affiliations:** 1 Clarity Clinical Research Consultants, New Zealand; 2 Computer Science Department, University of Otago, PO Box 56, Dunedin, New Zealand; 3 Department of Preventive and Social Medicine, University of Otago, PO Box 913, Dunedin, New Zealand; 4 Back In Motion Physiotherapy, Dunedin, New Zealand; 5 Physiotherapy Department, Dunedin Hospital, Otago District Health Board, Dunedin, New Zealand

## Abstract

**Background:**

Musculoskeletal physiotherapists routinely assess lumbar segmental motion during the clinical examination of a patient with low back pain. The validity of manual assessment of segmental motion has not, however, been adequately investigated.

**Methods:**

In this prospective, multi-centre, pragmatic, diagnostic validity study, 138 consecutive patients with recurrent or chronic low back pain (R/CLBP) were recruited. Physiotherapists with post-graduate training in manual therapy performed passive accessory intervertebral motion tests (PAIVMs) and passive physiological intervertebral motion tests (PPIVMs). Consenting patients were referred for flexion-extension radiographs. Sagittal angular rotation and sagittal translation of each lumbar spinal motion segment was measured from these radiographs, and compared to a reference range derived from a study of 30 asymptomatic volunteers. Motion beyond two standard deviations from the reference mean was considered diagnostic of rotational lumbar segmental instability (LSI) and translational LSI. Accuracy and validity of the clinical assessments were expressed using sensitivity, specificity, and likelihood ratio statistics with 95% confidence intervals (CI).

**Results:**

Only translation LSI was found to be significantly associated with R/CLBP (*p *< 0.05). PAIVMs were specific for the diagnosis of translation LSI (specificity 89%, CI 83–93%), but showed poor sensitivity (29%, CI 14–50%). A positive test results in a likelihood ratio (LR+) of 2.52 (95% CI 1.15–5.53). Flexion PPIVMs were highly specific for the diagnosis of translation LSI (specificity 99.5%; CI 97–100%), but showed very poor sensitivity (5%; CI 1–22%). Likelihood ratio statistics for flexion PPIVMs were not statistically significant. Extension PPIVMs performed better than flexion PPIVMs, with slightly higher sensitivity (16%; CI 6–38%) resulting in a likelihood ratio for a positive test of 7.1 (95% CI 1.7 to 29.2) for translation LSI.

**Conclusion:**

This study provides the first evidence reporting the concurrent validity of manual tests for the detection of abnormal sagittal planar motion. PAIVMs and PPIVMs are highly specific, but not sensitive, for the detection of translation LSI. Likelihood ratios resulting from positive test results were only moderate. This research indicates that manual clinical examination procedures have moderate validity for detecting segmental motion abnormality.

## Background

Musculoskeletal physiotherapists routinely assess lumbar spinal segmental motion and choose interventions on the basis of the findings of those assessments. However, the validity of clinical tests used to assess segmental motion has not been established. When physiotherapists examine the lumbar spine, common assessments include passive accessory intervertebral motion tests (PAIVMs) and passive physiological intervertebral motion tests (PPIVMs) [1,2]. Movement abnormalities, such as hypermobility, are believed to be detected by these assessments [1].

To date, the only evidence for the concurrent validity of manual testing for the presence of lumbar segmental instability (LSI) comes from two studies in which the presence of spondylolysis was considered a proxy for the presence of segmental hypermobility. The first comprised of a very small subgroup analysis (6 patients) of patients with spondylolysis, within a sample of 62 patients with non-specific LBP [3]. The results of that investigation indicated that PAIVMs and PPIVMs could identify the symptomatic level with 83% sensitivity and 98% specificity [3]. In the second study, manual assessment (combined information from both PPIVMs and PAIVMs) was 69% sensitive and 96% specific for detection of the lytic segment [4]. When analysis was restricted to subjects who reported visual analogue pain scores of greater than 4/10, sensitivity and specificity rose to 100% [4]. In addition, some preliminary evidence indicates that PAIVM testing may have predictive validity for the purpose of classifying patients in a 'stabilisation' category, who respond better to an exercise intervention intended to increase lumbar segmental stability [5].

As there is currently no evidence in the literature to establish the concurrent validity of manual therapy tests for the detection of excessive sagittal planar motion of the lumbar spine, the aims of this study were to estimate the accuracy of three common clinical assessment items for the detection of lumbar segmental hypermobility (PAIVMs, flexion PPIVMs, and extension PPIVMs), compared to a criterion standard of radiographic measurement of sagittal segmental rotation and translation.

## Methods

### Design

Physiotherapists with post-graduate training in musculoskeletal manual therapy recruited consecutive eligible patients presenting with a new episode of recurrent or chronic low back pain (R/CLBP). Recruiting took place in the physiotherapists' own clinics, between October 2001 and August, 2003. Patients were included if i) they presented with a new episode of low back pain and, ii) they had experienced similar low back pain before, the first episode of which was at least three months prior to the date of recruitment, or iii) they were experiencing persistent low back pain of at least three months duration. Patients were excluded if they i) had spinal surgery within the previous six months, or ii) had a history of traumatic fracture of the spine which resulted in permanent neurological deficit, iii) had a history of serious neurological or psychiatric disease, iv) were under 20 years of age, or v) were pregnant. This research was approved by the Otago and Canterbury Regional Ethics Committees (reference # 01/05/030 & 01/10/095) of the New Zealand Ministry of Health.

**Figure 1:**
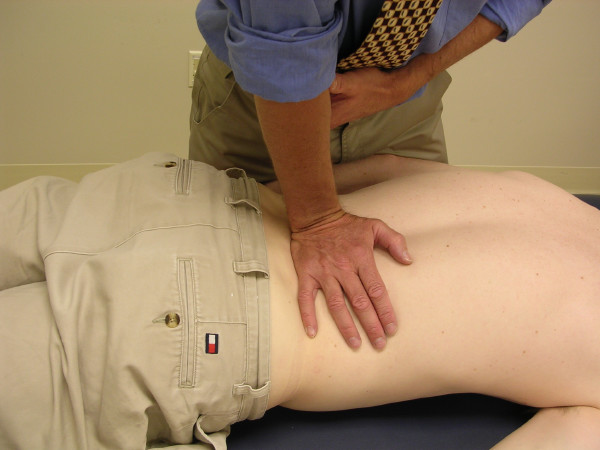
**The central posteroanterior passive accessory intervertebral motion (PAIVM) test**. The patient lies prone. The clinician contacts the spinous process of the target vertebra with the hypothenar eminence, and delivers a gradual posteroanteriorly directed force.

**Figure 2:**
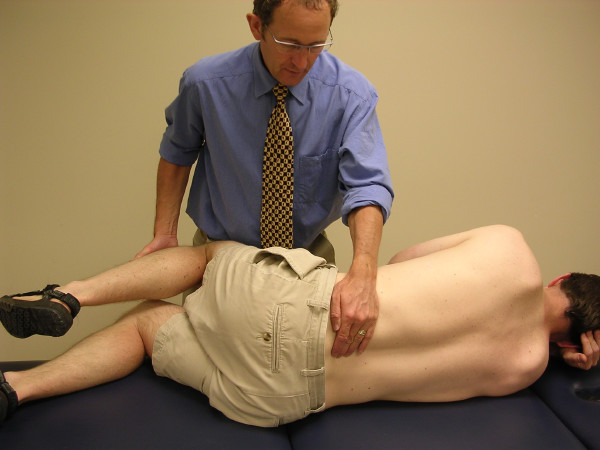
**The passive physiological intervertebral motion (PPIVM) test in flexion**. The patient is positioned side-lying. The clinician palpates the interspace between the adjacent spinous processes of the target motion segment with one finger, while moving the lumbar spine from neutral into flexion via the patient's uppermost limb.

**Figure 3:**
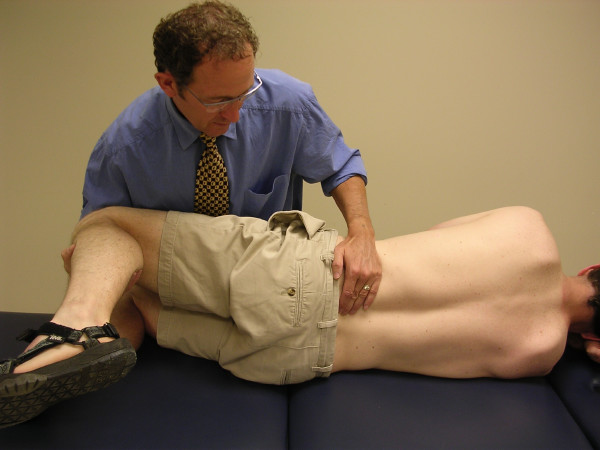
**The passive physiological intervertebral motion (PPIVM) test in extension**. The patient is positioned side-lying. The clinician palpates the interspace between the adjacent spinous processes of the target motion segment with one finger, while moving the lumbar spine from neutral to extension via the patient's uppermost limb.

The physiotherapists assessed PAIVMs and PPIVMs, at each lumbar segment, nested within a comprehensive physical examination. PAIVMs consisted of postero-anterior central pressure applied to the spinous processes, with the patient lying prone [1,2] (figure 1). PPIVMs were assessed with the patient side-lying, and consisted of moving the patients' spine through sagittal forward-bending (flexion) and backward-bending (extension), while palpating between the spinous process of adjacent vertebrae to assess the motion taking place at each motion segment [1,2] (figures 2 &3). PAIVM ratings were assessed on a 3 point ordinal scale, with 0 indicating hypomobility, 1 indicating normal motion, and 2 indicating hypermobility. PPIVMs were rated on a 5 point ordinal scale, with 0 & 1 indicating hypomobility, normal anchored at 2, and 3 & 4 indicating hypermobility. While pain responses were assessed, they were recorded separately from the assessment of motion, and were not included in the analysis for this study, which was concerned only with the assessment of spinal motion. Consenting patients were referred to radiology for flexion-extension lateral radiographs.

The reference standard for normal and abnormal spinal mobility measures was defined using the kinematic data from a sample of asymptomatic volunteers with no significant history of LBP, and no LBP within the prior three years. A sample of 30 asymptomatic adults was recruited and radiographed using the same protocol as the patient cohort. This project was approved by the University of Otago Human Ethics Committee.

For both cohorts, the sagittal rotation and translation motion of segments L2-3, L3-4, L4-5, and L5-S1 was measured using the method of Bodguk & Schneider [6-8], by researchers blinded to the clinical examination findings and radiologists' reports. Radiographs of insufficient quality to allow the analysis of two or more segments were excluded.

### Measurement procedures

Calculation of rotation and translation motion was performed using the Clarity*SMART *version 1.2 computer program [9]. Concurrent validity of rotation measurement by Clarity*SMART *v1.2 was tested against a reference standard (measurement using NIH Image [10]), and assessed using the intraclass correlation coefficient (ICC). Rotation measurement was tested against manual constructions (0.3 mm pencil on tracing paper; measurements using a 0.5 mm graduated ruler). These trials demonstrated near perfect concurrence for both rotation (ICC_(3,4) _of 0.98, 95% CI 0.92, 0.99), and translation (ICC_(3,1) _of 0.98, 95% CI 0.94, 0.99). Inter-rater reliability was excellent for both rotation (ICC_(3,1) _0.96, 95% CI 0.87, 0.99) and translation (ICC_(3,1) _0.83, 95% CI 0.46, 0.95).

### Data analysis

The reference standard for presence of LSI in the C/RLBP cohort was abnormal segmental hypermobility in excess of 2 standard deviations (sd) beyond the mean of a sample of 30 pain-free individuals. Prevalence of LSI findings in the C/RLBP cohort (i.e. the number of segments that fall beyond the 2sd cut-point derived from the kinematic data of the asymptomatic sample) were calculated. The chi squared (χ^2^) goodness of fit test was used to test the hypothesis that abnormal segmental hypermobility (i.e. LSI) is found in a higher proportion of patients with R/CLBP than would be expected in an asymptomatic sample. Significance was set at *p *< 0.05.

In concordance with the reference standard, only clinical PAIVM ratings of grade 2 and PPIVM ratings of grade 4 were considered positive for LSI. LSI was considered absent for all other data. For analysis of clinical examination data, both clinical and radiographic data were then collapsed into two regions, corresponding to upper lumbar and lower lumbar. This was decided *a priori*, and considered necessary because there is considerable evidence that therapists are not sufficiently accurate in identifying specific segmental levels by palpation, although they are usually within one level (up or down) and are generally reliable at locating again a segment they had previously located [11-13]. This inaccuracy presented an unacceptable risk of misclassification, that collapsing into regions would attenuate. Furthermore, it is also clear that some physical assessment procedures affect mobility at multiple segments [14] and that segmental specificity does not appear to be important with regard to application of physical therapies for LSI, including manual therapy [5,15-22] (although one study has found otherwise [23]). Data were thus collapsed into the 2 × 2 tables. By-segment results are, however, provided [see Additional file 1] for readers to compare.

Missing data resulted in list-wise deletion of the clinical and radiographic data, on a per-lumbar region, per-analysis basis. The accuracy of the clinical examination items was tested by calculating sensitivity and specificity from 2 × 2 contingency tables. Likelihood ratios were then calculated from these data. These statistics were calculated in Microsoft Excel, using a program written by the primary investigator (JHA). The program calculated 95% confidence intervals (CI) using Wilson's method for sensitivity & specificity, and the score method for likelihood ratios [24]. Methods and results were reported according to the STARD guideline checklist [25].

## Results

**Figure 4:**
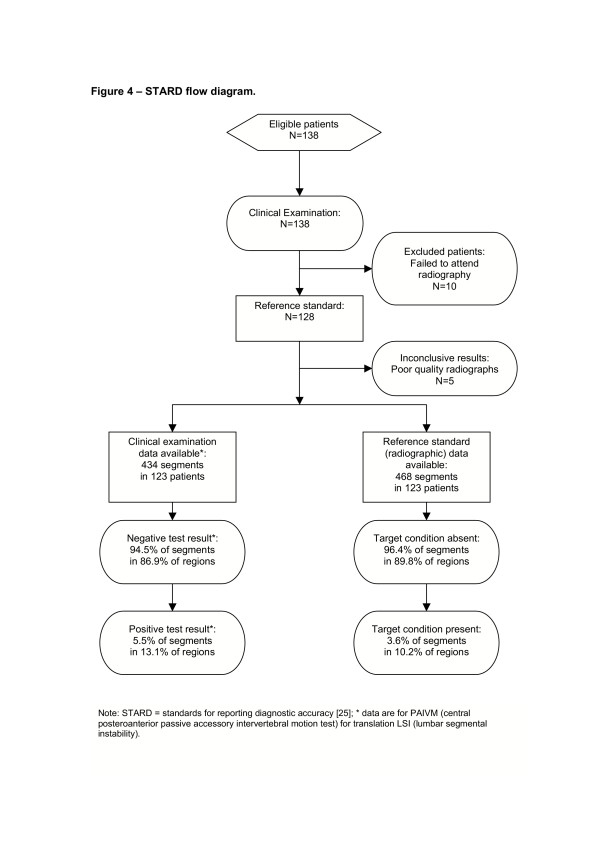
STARD flow diagram.

One hundred and thirty eight (138) consenting patients were recruited for clinical examination. One hundred and eight (108) were recruited in primary care; the remaining 30 presented to a hospital outpatient physiotherapy department. Ten patients failed to present to radiology for flexion-extension radiographs. Five sets of radiographs were of insufficient quality for analysis. Of the 123 included participants, 68 (55%) were males and 55 (45%) females. Further characteristics are described in Table 1. A STARD flow chart is provided in figure 4. No adverse events were reported.

**Table 1:** Description of the R/CLBP cohort

	Mean	sd	Range	N
Age	40.0	11.2	20–75	106
Body mass index	26.7	4.75	19.8–43.0	85
Years since first LBP episode	8.3	8.0	<1–33	104
Disability score (out of 18)	7.13	4.543	0–17	119
Pain level (out of 100)	42.7	25.7	0–100	117
Proportion with constant LBP	.23	.420	-	106
Proportion not working due to LBP	.12	.331	-	105
Delay between clinical examination and radiography (days)	5	5	-1 – 22	128

Notes: R/CLBP = recurrent or chronic low back pain. sd = Standard deviation; N = number with complete data. Disability score was assessed on the modified Roland-Morris RM18 [57]; Pain level was self-rated on a horizontal 10 cm visual analog scale.

Nine males and 24 females were available for recruitment into the asymptomatic sample. Three participants violated the exclusion criteria with regard to low back pain history, and were therefore ineligible. The asymptomatic sample therefore comprised of 9 males and 21 females, aged 23 to 60 years (mean 41.3, sd 12.8).

The 27 clinicians who collaborated on this study graduated with their first professional physiotherapy qualification between 1974 and 1996 (mean years since graduation 17, range 6 to 29). All had gained at least one post-graduate qualification in musculoskeletal physiotherapy which included training in manual therapy procedures for the spine, between 1983 to 2000 (mean years since graduation 8.7, range 2 to 19). They spent an average of 31 hours (interquartile range 21 to 40) per week treating patients, with LBP patients comprising, on average, 30% of their patient load (interquartile range 20 to 40).

### Prevalence of lumbar segmental instability

Sagittal rotation LSI was not found in statistically significant numbers (6 of 468 segments, or 1.3%), which is smaller than the number that would be expected by chance alone in a normally distributed sample of this size. Sagittal translation LSI was found at a prevalence of 3.6% (17 of 468 segments) (χ^2 ^p < 0.05). In this cohort, 5.6% of individuals had rotation LSI at least one segment, and 12.0% had translation LSI at least one segment.

### Accuracy of manual therapy assessment

PAIVMs and PPIVMs were specific for the diagnosis of both rotation LSI and translation LSI, but showed poor sensitivity. The accuracy statistics for PAIVM and PPIVM tests appear in Tables 2 &3. Full 2 × 2 contingency tables are also provided [see Additional file 1]. A positive PAIVM test (grade 2 on a scale from 0 to 2) results in likelihood ratios (LR+) of 2.74 and 2.52 for rotation LSI and translation LSI respectively. Extension PPIVMs performed better than flexion PPIVMs due to their slightly higher sensitivity. A positive extension PPIVM test (grade 4 on a scale from 0 to 4) results in LR+ of 8.4 and 7.1 for rotation LSI and translation LSI, respectively. Likelihood ratios for flexion PPIVMs were not statistically significant.

**Table 2:** Accuracy of PAIVMs for detecting lumbar segmental instability

LSI	Sensitivity (CI)	Specificity (CI)	LR+ (CI)	LR- (CI)
Rotation LSI	.33 (.12, .65)	**.88 (.83, .92)**	**2.74 (1.01, 7.42)**	.76 (.48, 1.21)
Translation LSI	.29 (.14, .50)	**.89 (.83, .93)**	**2.52 (1.15, 5.53)**	.81 (.61, 1.06)

Notes: PAIVMs = central posteroanterior passive accessory intervertebral motion tests; LSI = lumbar segmental instability; CI = 95% confidence interval; LR+ = likelihood ratio for a positive test; LR- = likelihood ratio for a negative test; Accuracy was assessed by lumbar region (upper lumbar and lower lumbar), overall results are presented; Items in bold type are statistically significant at *p *< 0.05.

**Table 3:** Accuracy of PPIVMs for detecting lumbar segmental instability

Flexion PPIVMs				
LSI	Sensitivity (CI)	Specificity (CI)	LR+ (CI)	LR- (CI)

Rotation LSI	.05 (.01, .36)	**.99 (.96, .1.00)**	4.12 (.21, 80.3)	.96 (.83, 1.11)
Translation LSI	.05 (.01, .22)	**.995 (.97, 1.00)**	8.73 (.57, 134.7)	.96 (.88, 1.05)

Extension PPIVMs				

LSI	Sensitivity (CI)	Specificity (CI)	LR+ (CI)	LR- (CI)

Rotation LSI	.22 (.06, .55)	**.97 (.94, .99)**	**8.40 (1.88, 37.55)**	.80 (.56, 1.13)
Translation LSI	.16 (.06, .38)	**.98 (.94, .99)**	**7.07 (1.71, 29.2)**	.86 (.71, 1.05)

Notes: PPIVMs = passive physiological intervertebral motion tests; LSI = lumbar segmental instability; CI = 95% confidence interval; LR+ = likelihood ratio for a positive test; LR- = likelihood ratio for a negative test; Accuracy was assessed by lumbar region (upper lumbar and lower lumbar), overall results are presented; Items in bold type are statistically significant at *p *< 0.05.

## Discussion

Despite their widespread use, the validity of PAIVMs and PPIVMs for assessing abnormal sagittal planar motion has not been previously established. We have found PAIVMs and PPIVMs to have high specificity, but poor sensitivity, for the diagnosis of both rotation LSI and translation LSI.

Like sensitivity and specificity, the likelihood ratio for a positive test (LR+) is more powerful when its value is high. Because of the many factors which must be taken into account when applying a diagnostic test to an individual patient (such as the setting the test is used in, purpose of applying the test, prevalence of the disorder, consequences of missing a diagnosis, and risk of harm from the indicated therapy), there are no set cut-off values for sensitivity, specificity, or likelihood ratios, however some authors provide general guidelines [26]. Tests returning LR+ values of 2 to 5 produce small but often useful changes in probability [26], while LR+ values of 5 to 10 (and greater) are more powerful. A test with a likelihood ratio of one is of no clinical utility. The results of this study indicate that a segment testing positive with a PAIVM test is approximately two-and-a-half times more likely to be hypermobile than not [27]. The results for PPIVMs were higher, indicating that a segment testing positive with an extension PPIVM test is approximately seven times more likely to be hypermobile than it is to be normal or hypomobile.

Likelihood ratios for negative tests from this research were less impressive than were the LR+ values, with values between 0.76 and 0.96. None were statistically significant. A LR- closer to zero is more powerful, whereas a LR- of one has no discriminative power. Tests returning LR- values of 0.2 to 0.5 produce small but useful changes in probability, while those with values less than 0.2 are more powerful [26]. This research indicates that a negative result for hypermobility with PAIVM or PPIVM tests is clinically uninformative.

The low prevalence of rotation LSI in this non-surgical, mostly primary care cohort indicate that sagittal rotation hypermobility does not appear to be associated with R/CLBP, as the number of segments hypermobile in rotation is less than the number that would be expected in a sample from a normally distributed asymptomatic population. Sagittal translation hypermobility was found in a significantly higher than expected proportion of patients with R/CLBP (12.0%), and therefore using a Gaussian definition of abnormality (i.e. beyond 2sd from a reference mean) [28] can be considered a valid clinical disorder. Only a small proportion of segments (3.6%) satisfied this Gaussian definition for sagittal translational LSI, however, indicating that it is neither common in this population nor strongly associated with C/RLBP. This may be considered surprising in the light of the emphasis on sagittal translation in the LSI literature [29,30]. This proportion does, however, compare well with clinicians' judgement using PAIVM tests. In the present study, therapists considered 5% of lumbar segments to have manual tests findings positive for LSI. This figure compares well to the 12% of patients with LBP reported to be hypermobile by therapists using PAIVM testing in other research [5]. With regard to the physical examination, though, it is also recognised that assessment of displacement kinematics alone may not be a sufficient basis for the diagnosis of LSI [31,32].

This study has a number of limitations which limit the interpretation of these results. Firstly, while the assessments were nested within a comprehensive clinical examination, and performed in the physiotherapists' own clinical setting, only these three physical assessments were studied in isolation. No attempt was made to identify clusters of assessments that may multiplicatively improve diagnostic accuracy. It is likely that these assessments would have much greater clinical utility within a cluster of other valid signs, symptoms, and history items [16,19]. Furthermore, it may be necessary to adjust the likelihood ratios of these and other tests researched in the future, to remove the influence of conditional dependence, using statistical methods such as logistic regression [33]. Secondly, the prevalence of LSI (using a Gaussian definition of abnormal motion) in this population is low. Defining LSI using a statistical model other than the Gaussian definition used here may result in different prevalence rates. We derived our cut-point for the definition of LSI from the results of our asymptomatic sample; validating the cut-points in another, independent sample would make these results more robust. Sensitivity and specificity, and hence likelihood ratios, may differ in a population with different prevalence rates, such as gymnasts or other athletes, patients with spondylolysis, or surgical candidates [34]. It is also well known that diagnostic tests achieve higher values in the secondary and tertiary care populations, where severity of disease is generally higher [34]. For this reason, too, values may differ in a population with a different spectrum of the target disorder(s), such as patients with spondylolisthesis or higher pain or disability scores. In the primary care low back pain population, the severity of low back conditions is generally low, making differential diagnosis more difficult. In the context of the present population, however, because mechanical low back pain is not life-threatening and the risks of physiotherapeutic interventions are very low [35], moderate index values are acceptable and may still be useful in the diagnosis of low back pain subgroups. Thirdly, analysis of segmental motion from flexion-extension radiographs was limited to sagittal segmental planar rotation and translation. These are properties of displacement kinematics, and as such identify only abnormalities in the quantity of motion. Other parameters of displacement kinematics, such as ratio of translation to rotation [36], instantaneous axis of rotation, and centre of reaction [6] may better characterise abnormalities of movement quality, rather than quantity. Motion abnormalities may also occur in the mid-range of movement and thus cannot be captured on flexion-extension radiographs, but may be detectable by videofluoroscopy. Furthermore, displacement kinematics are only one aspect of segmental motion (and may not be the most important aspect). The physical examination procedures employed by physiotherapists may assess important parameters other than displacement kinematics [32]. This study has not attempted to examine physical assessment of spinal motion velocity, acceleration, or temporal patterns of displacement, nor has it examined physical assessment of kinetics relevant to spinal segmental motion, such as stiffness, viscoelasticity, or force-displacement characteristics. Further research is warranted to fill in the gaps in the literature addressing these limitations.

This research has focussed on the diagnostic accuracy of PAIVMs and PPIVMs, and the multi-centre, pragmatic design of the study precluded assessment of their reliability. The reliability of these clinical assessments has been debated in the literature for many years [37,38]. While many studies have found reliability to be poor [39,40], others have reported considerably better reliability [41,42]. Contrary to popularly held opinion [43,44], it is not easy to conduct a valid and rigorous reliability study. The biostatistical literature points out quite clearly that there numerous difficulties and pitfalls to the study of reliability [45-52] which may threaten the validity of research results. Common methodological problems include violation of the assumptions necessary for the statistical tests used, selection of an inappropriate sample of subjects, lack of true variance in the levels or categories within the sample tested, low prevalence of results across the full spectrum of test scores, and skewed or assymetrical distribution of data. These factors all have a very large impact on the validity and interpretation of much of the literature available on the reliability of these physical examination items: much of the published research regarding reliability may be biased toward the null. It has been argued that tests can be useful for clinical decision-making, in spite of ostensibly low reliability [53], and that it is more important to establish validity of a test or measure [46]. For these reasons, it can be argued that reliability should only be studied in the context of validity [53]. Further research is warranted into these issues.

The first research published in the peer-reviewed literature to test the concurrent validity of these manual assessments for the detection of abnormal segmental rotation appeared in the literature only recently [54], and addressed lumbar segmental hypomobility. The findings of that research indicated that PAIVMs were moderately sensitive (75%) but not specific (35%) for the detection of hypomobility, while flexion PPIVMs were found to be specific (89%) but not sensitive (42%), with a LR+ of 3.9 [54]. Those findings, and others from the literature on predictive validity of hypomobility [16,55,56], are generally consistent with the present results, and represent a gathering body of evidence supporting the validity and clinical utility of these manual clinical assessments.

While the LR+ values reported in the present research are only of moderate strength, they may have some clinical utility. If a patient returns a positive test using the extension PPIVM, this would increase the probability that the lumbar segment being tested has translation LSI from 3.6% (the proportion of lumbar segments found to have LSI in this study) to 20.9%. Even assuming conditional independence of the tests, if the patient then returns a positive test using the central P-A PAIVM, post-test probability that the segment is hypermobile would rise to only 40%. This is, however, still too low for clinical or research usefulness, without further improvement in diagnostic certainty being available from other components of the clinical examination (such as the patients history and interview findings, other patient-derived information, and other physical signs). Research investigating the predictive validity of clinical examination findings has found that manual assessments of a similar nature to be a significantly useful addition to a clinical prediction rule, when combined in a test item cluster with other findings [16,55,56]. These factors mean that the LR+ values found in this study may be of a magnitude sufficient to be useful in clinical practice when combined with other information from the clinical examination.

## Conclusion

This study provides the first evidence reporting the concurrent validity of manual assessments for detecting the excessive sagittal planar motion associated with LSI *in vivo*. PAIVMs and PPIVMs were specific, but not sensitive, for the detection of rotation LSI and translation LSI. Positive PAIVM and extension PPIVM tests had statistically significant likelihood ratios for identifying translational LSI. The validity of the manual therapists' assessments of excessive sagittal planar motion was only moderate, but as these results do not take into account other important parameters of segmental mobility, such as stiffness or viscoelasticity, this level of validity is still encouraging. Further investigation into the validity of the clinical examination for the detection of lumbar segmental motion disorders is warranted, such as whether greater accuracy may be achieved from clinical examination when manual assessments are combined with other information from the patients' history and physical examination.

## Competing interests

The authors declare that they have no competing interests.

## Authors' contributions

JHA conceived, designed and coordinated the study, recruited the clinicians, recruited and examined some of the patients, carried out data analysis and prepared the manuscript. JHA retains copyright on all contents. BMcC assisted with measurement technology & data analysis, and manuscript preparation. PH provided statistical support. GM, CC, and TH assisted in clinician recruitment, patient recruitment and examination, data collection, and provided facilities. All authors read and approved the final manuscript.

## Pre-publication history

The pre-publication history for this paper can be accessed here:



## Supplementary Material

Additional file 1 Click here for file
