# Supplementary material for: Lumbar segmental instability: a criterion-related validity study of manual therapy assessment
Source: BMC Musculoskelet Disord. 2005 Nov 7;6:56. doi: 10.1186/1471-2474-6-56 (PMC1310529; doi:10.1186/1471-2474-6-56)
Supplement: Additional file 1 [file 1471-2474-6-56-s1.pdf]

Additional file 1

2x2 contingency tables of the results of the index (clinical) test by lumbar region, versus the results of the reference standard.

Accuracy of PAIVMs for detecting rotation LSI within a lumbar region (upper and lower lumbar regions)

	Reference standard +	Reference standard -	Row totals				
				Sensitivity (95%CI) =	0.333	(0.121	0.646)
Dx test +	3	24	27	Specificity (95%CI) =	0.878	(0.825	0.917)
Dx test -	6	173	179	LR - (95%CI) =	0.76	(0.48	1.21)
Column totals	9	197	206	LR+ (95%CI) =	2.74	(1.01	7.42)

Accuracy of Flexion PPIVMs for detecting rotation LSI within a lumbar region (upper and lower lumbar regions)

	Reference standard +	Reference standard -	Row totals				
				Sensitivity (95%CI) =	0.050	(0.005	0.345)
Dx test +	0.5*	2.5	3	Specificity (95%CI) =	0.988	(0.962	0.996)
Dx test -	9.5	203.5	213	LR - (95%CI) =	0.96	(0.83	1.11)
Column totals	10	206	216	LR+ (95%CI) =	4.12	(0.21	80.25)

Accuracy of Extension PPIVMs for detecting rotation LSI within a lumbar region (upper and lower lumbar regions)

	Reference standard +	Reference standard -	Row totals				
				Sensitivity (95%CI) =	0.222	(0.063	0.547)
Dx test +	2	5	7	Specificity (95%CI) =	0.974	(0.940	0.989)
Dx test -	7	184	191	LR - (95%CI) =	0.80	(0.56	1.13)
Column totals	9	189	198	LR+ (95%CI) =	8.40	(1.88	37.55)

Accuracy of PAIVMs for detecting translation LSI within a lumbar region (upper and lower lumbar regions)

	Reference standard +	Reference standard -	Row totals				
				Sensitivity (95%CI) =	0.286	(0.138	0.500)
Dx test +	6	21	27	Specificity (95%CI) =	0.886	(0.833	0.925)
Dx test -	15	164	179	LR - (95%CI) =	0.81	(0.61	1.06)
Column totals	21	185	206	LR+ (95%CI) =	2.52	(1.15	5.53)

Accuracy of Flexion PPIVMs for detecting translation LSI within a lumbar region (upper and lower lumbar regions)

	Reference standard +	Reference standard -	Row totals				
				Sensitivity (95%CI) =	0.045	(0.008	0.218)
Dx test +	1	1	2	Specificity (95%CI) =	0.995	(0.971	0.999)
Dx test -	21	191	212	LR - (95%CI) =	0.96	(0.88	1.05)
Column totals	22	192	214	LR+ (95%CI) =	8.73	(0.57	134.69)

Accuracy of Extension PPIVMs for detecting translation LSI within a lumbar region (upper and lower lumbar regions)

	Reference standard +	Reference standard -	Row totals				
				Sensitivity (95%CI) =	0.158	(0.055	0.376)
Dx test +	3	4	7	Specificity (95%CI) =	0.978	(0.944	0.991)
Dx test -	16	175	191	LR - (95%CI) =	0.86	(0.71	1.05)
Column totals	19	179	198	LR+ (95%CI) =	7.07	(1.71	29.24)

Notes: PAIVMs = central posteroanterior passive accessory intervertebral motion tests; PPIVMs = passive physiological intervertebral motion tests; LSI = lumbar segmental instability; Dx test = result of the clinical test; CI = 95% confidence interval; LR+ = likelihood ratio for a positive test; LR− = likelihood ratio for a negative test. Row and column totals differ from expected total (246) due to listwise deletion in the event of any missing values in either clinical or radiographic data. *0.5 added to all cells in the event of a zero in any cell.

2x2 contingency tables of the results of the index (clinical) test by lumbar segmental level, versus the results of the reference standard

Accuracy of PAIVMs for detecting rotation LSI by segment

	Reference standard +	Reference standard -	Row totals				
				Sensitivity (95%CI) =	0.167	(0.030	0.564)
Dx test +	1	23	24	Specificity (95%CI) =	0.946	(0.921	0.964)
Dx test -	5	405	410	LR - (95%CI) =	0.88	(0.62	1.26)
Column totals	6	428	434	LR+ (95%CI) =	3.10	(0.50	19.39)

Accuracy of Flexion PPIVMs for detecting rotation LSI by segment

	Reference standard +	Reference standard -	Row totals				
				Sensitivity (95%CI) =	0.071	(0.008	0.439)
Dx test +	0.5*	2.5	3	Specificity (95%CI) =	0.994	(0.982	0.998)
Dx test -	6.5	442.5	449	LR - (95%CI) =	0.93	(0.76	1.15)
Column totals	7	445	452	LR+ (95%CI) =	12.71	(0.67	241.27)

Accuracy of Extension PPIVMs for detecting rotation LSI by segment

	Reference standard +	Reference standard -	Row totals				
				Sensitivity (95%CI) =	0.071	(0.008	0.439)
Dx test +	0.5*	4.5	5	Specificity (95%CI) =	0.989	(0.974	0.996)
Dx test -	6.5	409.5	416	LR - (95%CI) =	0.940	(0.76	1.15)
Column totals	7	414	421	LR+ (95%CI) =	6.75	(0.39	110.76)

Accuracy of PAIVMs for detecting translation LSI by segment

	Reference standard +	Reference standard -	Row totals				
				Sensitivity (95%CI) =	0.188	(0.066	0.430)
Dx test +	3	21	24	Specificity (95%CI) =	0.950	(0.924	0.967)
Dx test -	13	397	410	LR - (95%CI) =	0.86	(0.68	1.08)
Column totals	16	418	434	LR+ (95%CI) =	3.73	(1.24	11.23)

Accuracy of Flexion PPIVMs for detecting translation LSI by segment

	Reference standard +	Reference standard -	Row totals				
				Sensitivity (95%CI) =	0.028	(0.003	0.219)
Dx test +	0.5*	2.5	3	Specificity (95%CI) =	0.994	(0.982	0.998)
Dx test -	17.5	431.5	449	LR - (95%CI) =	0.98	(0.90	1.06)
Column totals	18	434	452	LR+ (95%CI) =	4.82	(0.24	96.82)

Accuracy of Extension PPIVMs for detecting translation LSI by segment

	Reference standard +	Reference standard -	Row totals				
				Sensitivity (95%CI) =	0.033	(0.003	0.253)
Dx test +	0.5*	0.5	1	Specificity (95%CI) =	0.999	(0.988	1.000)
Dx test -	14.5	401.5	416	LR - (95%CI) =	0.97	(0.88	1.06)
Column totals	15	402	417	LR+ (95%CI) =	26.8	(0.55	1305.5)

Notes: PAIVMs = central posteroanterior passive accessory intervertebral motion tests; PPIVMs = passive physiological intervertebral motion tests; LSI = lumbar segmental instability; Dx test = result of the clinical test; CI = 95% confidence interval; LR+ = likelihood ratio for a positive test; LR− = likelihood ratio for a negative test. Row and column totals differ from expected total (246) due to listwise deletion in the event of any missing values in either clinical or radiographic data. *0.5 added to all cells in the event of a zero in any cell.
